# Targeted delivery of oligonucleotides using multivalent protein–carbohydrate interactions

**DOI:** 10.1039/d2cs00788f

**Published:** 2023-01-27

**Authors:** Vajinder Kumar, W. Bruce Turnbull

**Affiliations:** a Department of Chemistry, Akal University, Talwandi Sabo Bathinda Punjab India vkvkumar17@gmail.com; b School of Chemistry and Astbury Centre for Structural Molecular Biology, University of Leeds Leeds UK W.B.Turnbull@leeds.ac.uk

## Abstract

Cell surface protein–carbohydrate interactions are essential for tissue-specific recognition and endocytosis of viruses, some bacteria and their toxins, and many glycoproteins. Often protein–carbohydrate interactions are multivalent – multiple copies of glycans bind simultaneously to multimeric receptors. Multivalency enhances both affinity and binding specificity, and is of interest for targeted delivery of drugs to specific cell types. The first such example of carbohydrate-mediated drug delivery to reach the clinic is Givosiran, a small interfering ribonucleic acid (siRNA) that is conjugated to a trivalent *N*-acetylgalactosamine (GalNAc) ligand. This ligand enables efficient uptake of the nucleic acid by the asialoglycoprotein receptor (ASGP-R) on hepatocytes. Synthetic multivalent ligands for ASGP-R were among the first ‘cluster glycosides’ developed at the birth of multivalent glycoscience around 40 years ago. In this review we trace the history of ‘GalNAc targeting’ from early academic studies to current pharmaceuticals and consider what other opportunities could follow the success of this delivery technology.

## Abstract 2

This review traces the development of targeted delivery of nucleic acids to liver cells using multivalent carbohydrate ligands.

## Graphical abstract

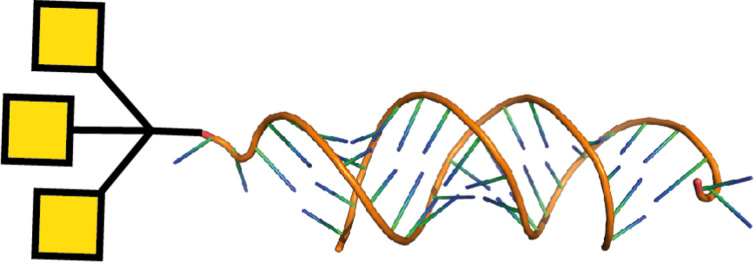

## Introduction

**Fig. 1:**
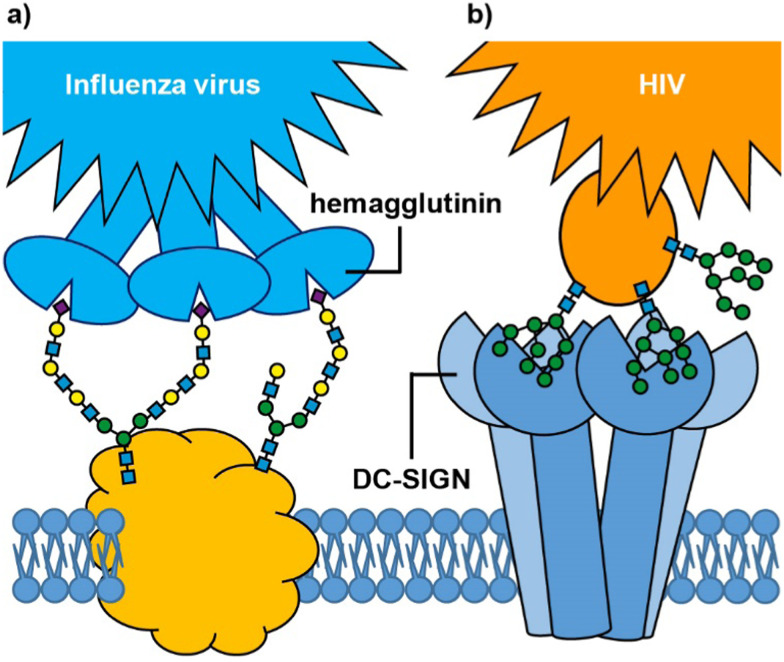
Glycan-mediated cell targeting: (a) influenza virus hemagglutinin binds to multiple sialic acids on cell surface glycoproteins; (b) cell surface DC-SIGN receptor binds to mannosylated glycans on the surface of HIV.

Many biological processes depend on interactions between cell surface carbohydrates (glycans) and carbohydrate-binding proteins (lectins).^1,2^ These functions include normal physiological processes such as modulation of immune activity,^3^ fertilization,^4,5^ and cell signalling.^6^ However, lectins also mediate cell adhesion and entry by viruses, bacteria and bacterial toxins.^7^ In some cases, it is a glycan on the cell surface that binds to an exogenous lectin, while in others it is a cell surface lectin that acts as the receptor for an exogenous glycoconjugate; for example, influenza virus uses its hemagglutinin lectins to bind to cell surface sialylated glycans in the respiratory tract (Fig. 1(a)),^8,9^ whereas HIV enters dendritic cells when the virus glycoproteins bind to the DC-SIGN cell surface receptor (Fig. 1(b)).^10^ Irrespective of whether it is the glycan or lectin that is displayed on the cell surface, protein–carbohydrate interactions can provide exquisite selectivity to target specific cells/tissues.^11^

An important feature of protein–glycan interactions is multivalency. The binding of individual monosaccharides to a protein is often weak, so natural lectin–glycan interactions typically combine multiple, simultaneous weak interactions to give rise to the strong, yet reversible, non-covalent binding that is necessary to perform important biological functions.^12^ Sometimes this involves lectins with multiple copies of the binding site (Fig. 1), while in other cases it may be multiple copies of a monovalent lectin on a cell surface. In addition to enhancing binding affinity,^13^ multivalency can also greatly enhance binding selectivity. So-called superselective binding arises above a threshold density of the target ligand on the cell surface,^14,15^ which opens the possibility of discriminating between target cell types that have different quantities of a particular glycan or lectin displayed on their surfaces. Multivalency has also been found to influence the mechanism by which species enter cells. Two major routes for endocytosis are the caveolae- and clathrin-mediated pathways.^16^ Nanoparticles with low loading of ligands favour caveolae-mediated endocytotic pathways, which can lead the nanoparticles to the compartments surrounding the nucleus (Golgi and endoplasmic reticulum), while avoiding lysosomes where molecules are degraded.^17^ In contrast, nanoparticles with high valencies predominantly enter cells by clathrin-mediated pathways, which traffics them towards lysosomal degradation.

The principles of multivalency have been appreciated since the 1970s when Hornick and Karush,^18^ and Ehrlich^19^ cast light on the importance of multivalency in antibody affinity to proteins and cell–cell interactions through the principle of avidity and specificity. However, since the seminal work by Lee and co-workers on the synthesis and application of unnatural multivalent glycoconjugates,^20–22^ the field of multivalent glycoscience has grown rapidly to exploit what Lee and Lee called the ‘cluster glycoside effect’.^23–25^ While many studies on multivalent glycoconjugates have focussed on discovering inhibitors of protein–glycan interactions,^12,26^ their use for cell targeting dates back to the early studies of Lee and co-workers on binding and internalisation of cluster glycosides by hepatocytes *via* the asialoglycoprotein receptor.^21^ These seminal studies laid the foundation for the development of the first clinical application of synthetic multivalent carbohydrates. In 2019, the US Food and Drug Administration (FDA) approved Givosiran,^27^ a small interfering RNA (siRNA) drug that is targeted to hepatocytes by a trivalent carbohydrate ligand based on *N*-acetylgalactosamine (GalNAc) for the treatment of acute hepatic porphyria.^28^ A siRNA is a short double stranded RNA molecule that can be used to suppress gene expression in a sequence-specific manner through the process of RNA interference (RNAi).^29^ One of the RNA strands is known as the guide strand and has a complementary sequence to the mRNA to be degraded (*i.e.* the guide is the antisense sequence for the target mRNA). When the guide strand becomes incorporated into the RNA-induced silencing complex (RISC) it can then bind and destroy the target mRNA, thus preventing expression of its encoded protein.

Earlier oligonucleotide precision drugs were hailed as game-changing innovations with enormous commercial potential,^30^ but suffered from drawbacks such as extracellular/intracellular enzymatic degradation, unwanted side effects, rapid renal clearance, sequestration of plasma proteins, activation of the immune system, and low specificity.^29,31–34^ As a result, their potential for clinical efficacy was low. The advent of GalNAc technology helped to convert the promises of oligonucleotide drugs into clinical reality.^35^ While development of successful nucleic acid therapeutics has also required many advances in hydrolytically-stable analogues of phosphodiester linkages, this field has been reviewed in detail elsewhere,^36,37^ and will not be described here. In this tutorial review, we will focus on the early fundamental studies on the asialoglycoprotein receptor which underpinned the development of GalNAc-targeted RNA therapeutics. We will then consider recent developments and future opportunities for exploiting carbohydrate–protein interactions for targeting other tissue types.

### The asialoglycoprotein receptor (ASGP-R)

The liver is a chemical factory that performs many biological functions.^38^ It metabolises carbohydrates, proteins and fats; stores glycogen and vitamins; produces several plasma proteins involved in blood clotting; metabolises drugs and toxins; clears aging blood cells and plasma proteins from circulation. In the course of their day-to-day function, liver cells (hepatocytes) get frequent exposure to drugs, toxic materials and microbes that cause various liver-oriented diseases. Therefore, targeted delivery of drugs to hepatocytes is of particular importance.

**Fig. 2:**
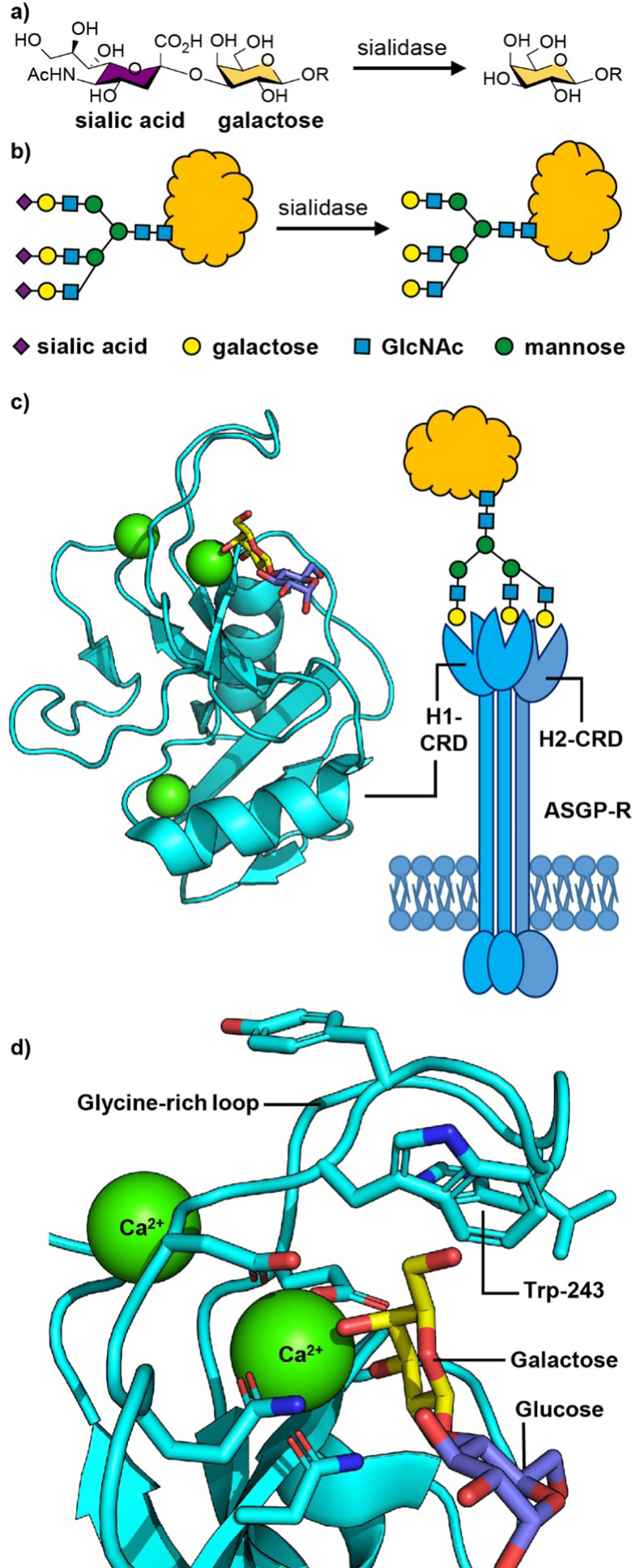
(a) Chemical structure and (b) cartoon structure of 3′-sialyl galactosyl groups on glycoproteins that lose sialic acid residues through action of sialidase enzymes leading to asialoglycoproteins. (c) The asialoglycoprotein receptor (ASGP-R) and crystal structure of the carbohydrate recognition domain (CRD) of the human ASGP-R bound to lactose; the three bound calcium ions are shown as green spheres. (d) Expansion of the galactose binding site showing the interaction between O-3 and O-4 of galactose with a calcium ion and the glycine-rich loop and tryptophan-243 residue that define the galactose/GalNAc binding selectivity. Figure created in PyMOL using 5jpv.pdb.

The mechanism by which hepatocytes recognise and remove aging proteins from blood relies on detecting changes in the carbohydrates that are attached to the circulating proteins. Sialic acids, the most common of which is *N*-acetylneuraminic acid (Neu5Ac),^39^ are commonly found as terminal sugar residues in human glycoproteins (Fig. 2(a) and (b)), in which they are attached to galactose (Gal) and sometimes *N*-acetylgalactosamine (GalNAc) residues.^40^ In the late 1960s, Ashwell and colleagues identified evidence for the presence of a receptor in the liver that could rapidly remove glycoproteins lacking terminal sialic acid residues.^41^ This asialoglycoprotein receptor (ASGP-R), first isolated in 1974 was shown to be a galactose-binding protein, and is recognised as being the first mammalian lectin to be discovered.^42,43^ ASGP-R is one of the most prominent and dominant C-type (calcium-dependent) lectins present on the sinusoidal, basolateral membrane of hepatic liver cells where it is present in very high density (up to 500 000 receptors per hepatocyte).^44,45^

The human ASGP-R is composed of two distinct protein subunits (Fig. 2(c)): H1 is the major subunit and H2 is the minor subunit, which can also exist in a H3 form which only differs from H2 only in its glycosylation.^46^ The H1 chain comprises a cytoplasmic N-terminal domain (40 amino acids), a transmembrane domain (20 amino acids), an extracellular stalk domain (80 amino acids) and a C-type lectin carbohydrate recognition domain (CRD; 140 amino acids). The H2 variant is slightly larger with the main difference being an additional 18 amino acid insert in the cytoplasmic domain. The ASGP-R is functionally multimeric with most models suggesting a 2 : 1 complex of H1 and H2,^46,47^ however higher order oligomers have also been proposed.^48,49^

The carbohydrate recognition domain (CRD) has a typical C-type lectin fold and three binding sites for calcium ions, one of which coordinates to O-3 and O-4 of the galactose or GalNAc ligand (Fig. 2(c)).^50^ The binding specificity for galacto-configured sugars is defined by the two residues that coordinate the calcium ion and a glycine-rich loop that allows tryptophan-243 to stack against the hydrophobic face of the Gal/GalNAc residue (Fig. 2(d)). A molecular dynamics study of an ASGPR-triantennary glycan complex (Fig. 2(c)) by Ramadugu *et al.* indicated that it is possible for the three CRDs to bind simultaneously to a single triantennary glycan through a chelation mechanism.^47^ In this configuration, the binding sites are oriented toward each other in an asymmetric arrangement that matches the distances between the terminal galactosyl residues in the glycan (20.9 ± 1.3, 12.5 ± 1.4, and 23.3 ± 3.1 Å).

The chelation mechanism hypothesis is also supported by the substantial step-wise increases in inhibitory potency (*ca.* 440-fold) on moving from mono- to biantennary, and from bi- to triantennary glycans.^51^ The triantennary glycan in Fig. 2(c) is 195 000 times more potent as an inhibitor of ASGPR binding than a similar compound with a single galactosyl group.^51^ In contrast, a triantennary high mannose glycan (as shown in Fig. 1(b)) is only 37-fold more potent as an inhibitor of DC-SIGN binding than a monovalent mannoside.^52^ In the latter case the distance separating the CRD binding sites is *ca.* 40 Å,^53^ which is too far to be spanned by a single glycan. NMR spectroscopy studies have shown that calcium binding, and hence glycan binding, is dependent on both calcium ion concentration and pH.^54^ The implication is that as the pH decreases in early endosomes, the apo-form of the CRD (*i.e.* calcium-free) become dominant, thus releasing the ligand from the ASGP-R.

### Early studies in the design of multivalent ligands for ASGP-R

**Fig. 3:**
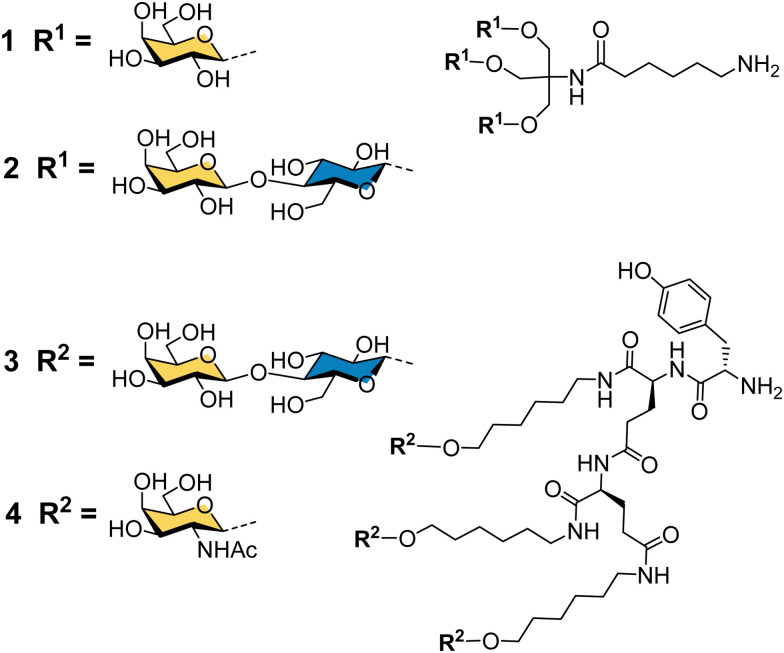
Cluster glycoside ligands for ASGP-R developed by Lee and co-workers.

Binding specificity studies in the 1980s laid the design rules for effective binding to ASGP-R. Baenziger and Maynard identified the preference of ASGP-R for GalNAc over galactose during studies of glycosidase-treated glycoproteins.^55^ Y. C. Lee and co-workers found that the GalNAc monosaccharide was 19-fold better than galactose as an inhibitor of the asialoorosomucoid glycoprotein binding to isolated ASGP-R, and 27-fold better for inhibition of the glycoprotein binding to cultured hepatocytes.^21^ These observations later proved to be very important for the development of the highest affinity synthetic ligands for ASGP-R. Lee had previously developed the first cluster glycosides based on a trivalent core derived from the common tris buffer (2-amino-2-(hydroxymethyl)-1,3-propanediol).^20^ The trivalent galactosyl 1 and lactosyl 2 clusters (Fig. 3) were up to 100-fold more potent inhibitors than monovalent analogues, and were internalized by cells upon binding.^21^ Trivalent lactoside 2 showed the lowest IC_50_ (concentration for 50% inhibition of asialoorosomucoid binding to ASGP-R) of 400 nM, however, their studies with synthetic *N*-linked glycans,^51^ indicated that triantennary glycans could achieve even lower IC_50_ values on the order of 1 nM. Their second generation compounds based on a tyrosine–glutamate–glutamate (YEE) tripeptide scaffold allowed greater spacing between the lactosyl units and a 10-fold further increase in potency for compound 3 compared to the tris-based lactosyl cluster 2.^22^ By comparing a series of natural and synthetic branched glycans, they concluded that the likely distances between ASGP-R binding sites would be on the order of 15, 22 and 25 Å.^56^ This estimate from 1984 is remarkably close to the 2010 estimate from molecular dynamics calculations of 20.9, 12.5 and 23.3 Å,^47^ and was a significant step in defining design criteria for multivalent ligands for ASGP-R. In 1987, Lee and Lee showed a trivalent GalNAc ligand 4 based on their YEE glycopeptide scaffold was about 1000-fold more potent than the corresponding lactosyl cluster for inhibiting binding to isolated rat ASGP-R, and achieved a 0.2 nM IC_50_ for inhibiting asialoorosomucoid binding to rat hepatocytes.^57^ This optimised Tri-GalNAc ligand 4 was later used by several research groups for targeted delivery of nucleic acids,^58–61^ and the anti-malarial drug primaquine,^62^ to hepatocytes *in vitro* and *in vivo*.

### Targeted delivery of nucleic acids to hepatocytes using ASGP-R ligands

**Fig. 4:**
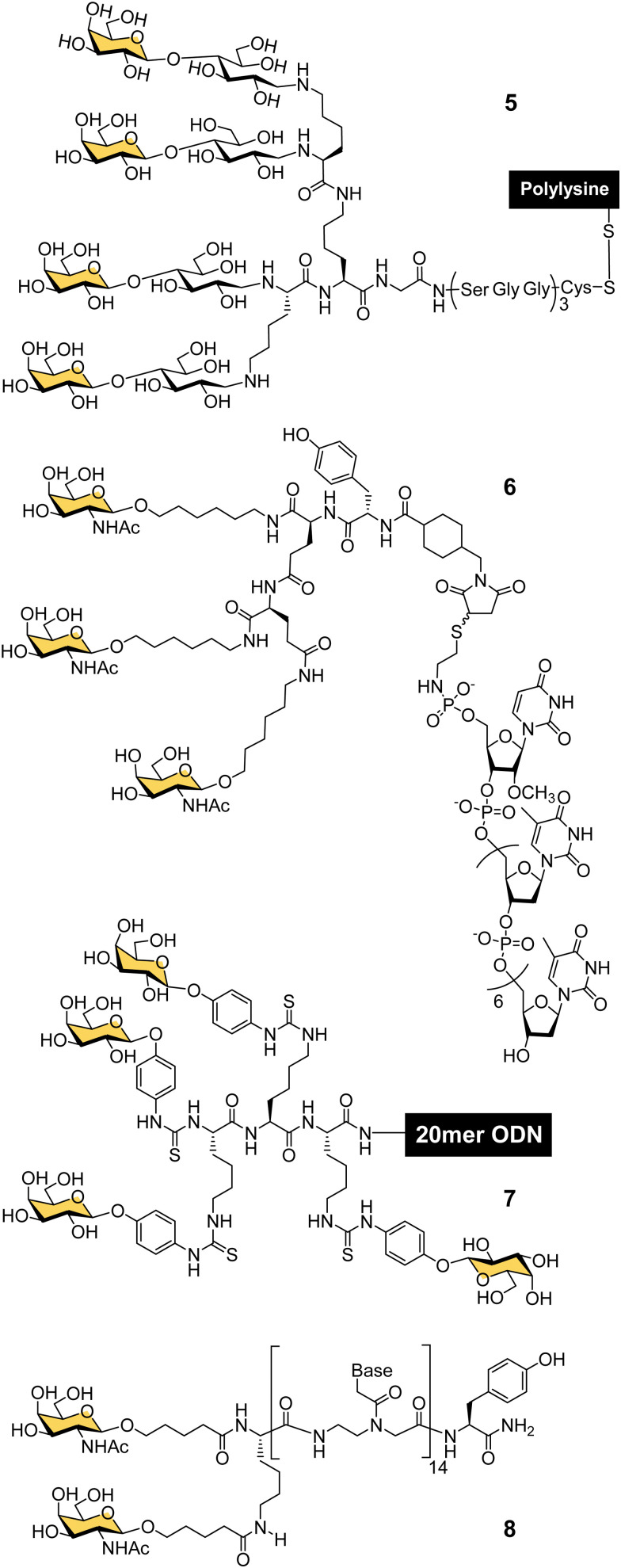
Gal- and GalNAc-clusters for oligonucleotide delivery.

In the late 80s and early 90s, Wu and Wu first demonstrated that nucleic acids could be targeted to hepatocytes using ligands for ASGP-R. They made a conjugate of asialoorosomucoid and polylysine and demonstrated that it could form a complex with plasmid DNA for selective transfection of cultured HepG2 human hepatocellular carcinoma cells,^63,64^ and rat hepatocytes *in vivo*.^65^ They also showed that a 21-mer hydrolytically stable phosphorothioate oligodeoxynucleotide (ODN), with a complementary sequence to the polyadenylation signal for hepatitis B virus (HBV) was a functionally active agent that could be delivered into cultured HepG2 cells and reduce expression of HBV surface antigen by 80% after 24 hours. Wagner and co-workers were able to replace the glycoprotein-based targeting group with a synthetic tetravalent glycopeptide (5) derived from lactose (Fig. 4), to which they conjugated polylysine and showed transfection of HepG2 cells with a gene for luciferase.^66^ A group from TargeTech Inc. achieved a similar result with polylysine conjugated to Lee's YEE(ahGalNAc)_3_ ligand 4.^58^

While conjugates of ASGP-R ligands with polylysine proved effective for nucleic acid delivery, such systems derived from synthetic polymers are inevitably heterogeneous mixtures. The next significant development came from a collaboration between Lee and Ts’o who first reported a structurally defined conjugate of an oligonucleotide and trivalent GalNAc targeting ligand in 1995.^59^ They combined a YEE(ahGalNAc)_3_ ligand that was derivatised with a maleimide group and a thiol-functionalised ODN methylphosphonate (pU^m^pT_7_) to make conjugate 6. The compound was selectively taken up by cells expressing ASGP-R,^59^ leading to at least a 20-fold increase in intracellular oligonucleotide concentration.^60^ An antisense oligonucleotide against HBV was effectively delivered into cultured HepG2 cells using the YEE(ahGalNAc)_3_ ligand, and led to 90% reduction of viral DNA expression at a concentration (1 μM) where the non-conjugated ODN had no effect.^60^ In mouse experiments, up to 70% of the GalNAc conjugate was rapidly transferred from the blood stream to the liver within 15 minutes of intravenous injection.^60^ Biessen, van Berkel and co-workers were also actively developing structurally-defined galactosylated ODNs and peptide nucleic acids (PNAs) in this period. They observed similarly efficient uptake to the liver of a tetravalent galactosyl ODN 7 in rats,^67^ and about 50% uptake to liver cells after 10 min for a divalent GalNAc–PNA 8.^68^ Delivery of an antisense PNA using this di-GalNAc approach enabled downregulation of microsomal triglyceride protein by 70% in freshly isolated mouse parenchymal liver cells.^69^

### Tris-based glycoclusters for targeting HDL/LDL to the liver

**Fig. 5:**
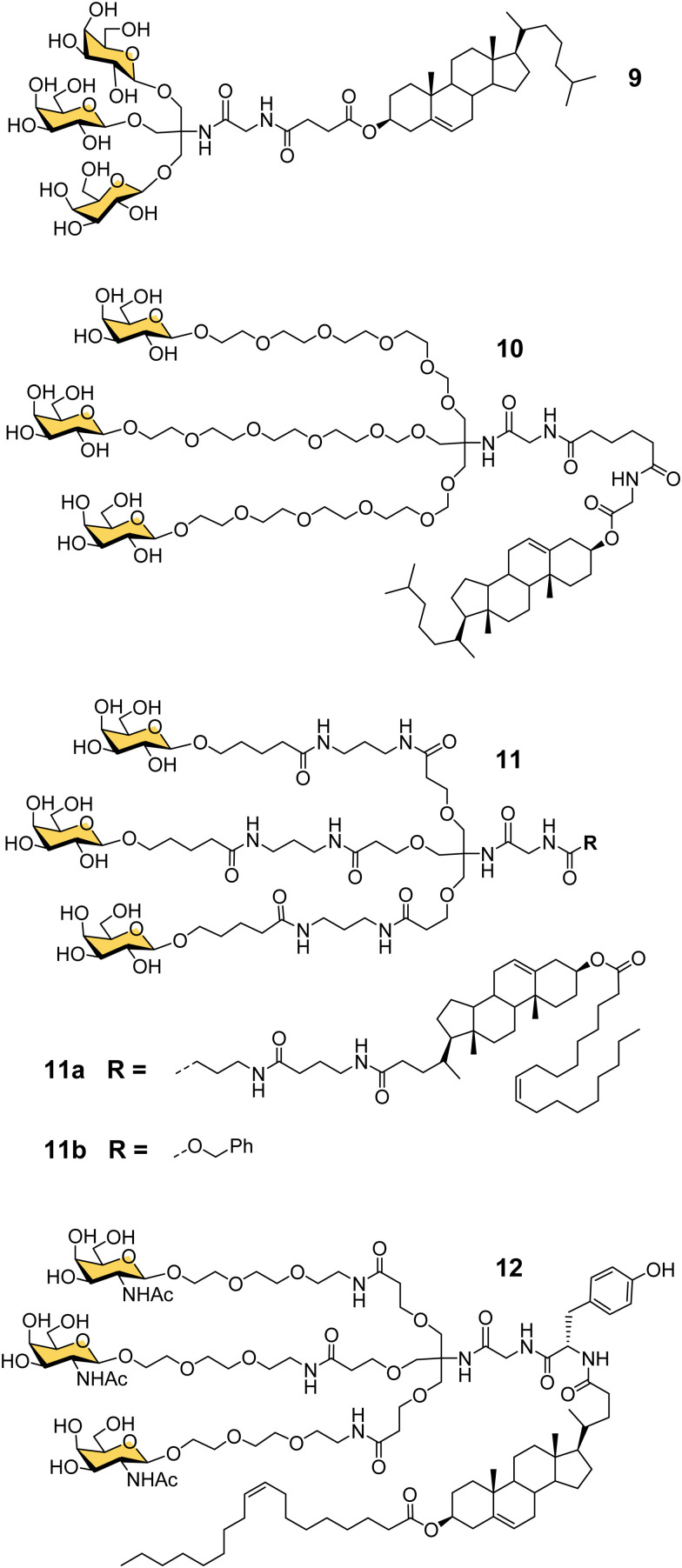
Tris-based glycoclusters for targeting HDL/LDL to the liver.

Another important contribution to the development of GalNAc-mediated ODN delivery came from a parallel series of studies on targeted delivery of high and low density lipoprotein (HDL/LDL) to parenchymal liver cells. In the mid-1980s van Berkel reported a trivalent galactoside conjugated to cholesterol that could target high density lipoproteins (HDL) to liver hepatocytes *in vivo*, which could enable lowering of serum cholesterol levels.^70,71^ The trivalent glycocluster 9 (Fig. 5) was constructed using the same tris buffer-derived scaffold used by Lee in his original cluster glycosides.^20^ With an aim to improve the binding efficiency of the tris-galactose clusters, Biessen, van Berkel and co-workers designed a series of trivalent galactosides with increasing lengths of ethylene glycol-based linkers between the sugar and tris core.^72^ They found that compound 10, which has a 20 Å spacer between the galactose residue and the branching point, had at least 2000-fold increase in activity over the trivalent compound lacking a spacer. This result suggests that optimal binding requires a longer interglycosidic distance of *ca.* 32 Å than the previously calculated binding site distance of 15–25 Å,^56^ but it is probably unlikely that the oligoethyleneglycol linkers would all adopt a fully extended conformation in solution. The design was further refined to improve its stability, and reduce its hydrophilicity as high water solubility of the lipid-linked compound 10 had limited its effectiveness as a multivalent glycolipid for directing lipoproteins to the liver.^73^ The result was trivalent galactoside 11a, for which a non-lipidated version (11b) displayed a *K*_i_ of 93 nM for hepatocyte binding. However, a potential problem for all multivalent glycoconjugates is cross-reactivity with other carbohydrate-binding proteins, and in this case higher concentrations of glycolipid 11a led to uptake by the macrophage galactose receptor (MGR) on Kupffer cells in the liver rather than ASGP-R on hepatocytes.^74^ Iobst and Drickamer have shown that the ASGP-R and MGR bind to multivalent galactosylneoglycoproteins with similar affinity, but while MGR shows no selectivity between Gal and GalNAc, ASGP-R was found to have a 60-fold preference for binding to GalNAc,^75^ albeit this number is higher than reported in other studies.^21,54^ Rensen *et al.* produced the GalNAc glycolipid 12, and achieved selective uptake of HDL and LDL particles by hepatocytes, and a lowering of blood cholesterol in a mouse model of familial hypercholesterolemia.^76^ While the ligands in this section were principally designed for HDL/LDL targeting, a GalNAc analogue of glycocluster 11 has proven central to the development of GalNAc-mediated targeting of ODNs.

### Taking GalNAc targeting to the clinic

The studies described thus far played a significant role in defining the criteria for designing well-defined multivalent ligands for ASGP-R that have high affinity and selectivity, considering the impact of the number and type of sugar residues, their spatial orientation, length and nature of the linker (*e.g.* hydrophilic–hydrophobic balance).^77^ By the turn of the century several groups had reported synthetic multivalent Gal and GalNAc ligands that could achieve functional delivery of ODNs or PNAs into cultured cells, and their distribution, *in vivo*, in rodents.^58,60,66,69^ However, that was still a long way from clinical application, which is a difficult challenge for academic groups to achieve alone. As Biessen and van Berkel note in a recent review highlighting their contributions to the development of GalNAc delivery,^78^ while they had patented their ASGP-R ligands in the mid-1990s, without a major pharmaceutical partner on board, the patent had expired. However, the interest of Dr Muthiah Manoharan and his team at Alnylam Pharmaceuticals (founded in 2002) in targeted delivery of RNAi drugs,^78^ enabled the translation of GalNAc targeting to the clinic.

**Fig. 6:**
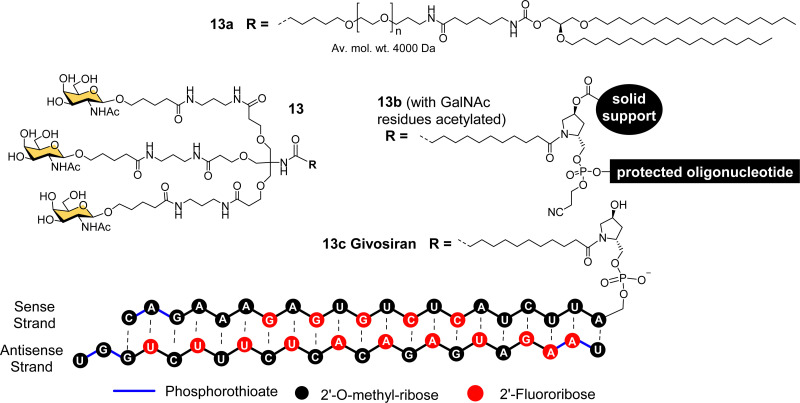
Chemical structure of triantennary GalNAc ligand 13 developed by Alnylam Pharmaceuticals for lipid conjugates (13a), solid-supported oligonucleotide synthesis (13b) and siRNA drug Givosiran (13c).

Considering all the factors for making the best multivalent ligand, Alnylam Pharmaceuticals developed a high affinity (*K*_d_ = 2.3 nM) trivalent glycocluster ligand (Fig. 6, 13) for ASGP-R, closely based on compound 11 but with GalNAc sugars rather than galactose.^79^ A lipidated version of this ligand (13a) with a PEG spacer was effective for delivering short interfering RNA (siRNA) molecules in lipid nanoparticles to reduce expression of the serum protein factor VII in mice.^80^ Alnylam Pharmaceuticals also developed a version of the ligand with a *trans*-4-hydroxyprolinol linker (13b) that allowed attachment of the protected GalNAc cluster to a solid support for construction of oligonucleotides by solid phase synthesis.^81^ A GalNAc-targeted siRNA synthesised in this way gave effective suppression of transthyretin expression in the liver following sub-cutaneous injection in mice.^81^

Acute hepatic porphyrias (AHP) are inherited disorders resulting from deficiencies in haem biosynthesis.^28^ Elevated expression of the first enzyme in the haem biosynthetic pathway, 5-aminolevulinic acid synthase (ALAS1), results in accumulation of neurotoxins 5-aminolevulinic acid (ALA) and porphobilinogen (PBG). Alnylam Pharmaceuticals used their GalNAc ligand to produce a siRNA drug to suppress ALAS1 expression.^82^ The compound was effective in rodent and non-human primate experiments when administered sub-cutaneously, giving rise to reduced ALAS1 mRNA levels in liver and lower levels of ALA and PBG in plasma and urine.^82^ This compound, named Givosiran (13c), entered a phase I clinical trial in 2015, a phase I/II trial in 2016, and a phase III trial in 2017.^27,83,84^ In 2019, US Food and Drug Administration (FDA) approved this first siRNA GalNAc-based drug Givosiran, for the treatment of AHP, and it received a positive opinion from the European Medicines Agency (EMA) in 2020.^27,83^

Recently, two more drugs, Inclisiran^85^ and Lumasiran,^86^ have also been approved by the US FDA and EMA to treat hypercholesterolemia and primary hyperoxaluria type 1 (PH1), respectively. Two other GalNAc–siRNA conjugates from Alnylam Pharmaceuticals, Vutrisiran and Fitusiran, showed convincing results in the phase III clinical trials. Vutrisiran (ALN-TTRsc02) is an investigational RNAi therapeutic in development to treat transthyretin-mediated (ATTR) amyloidosis.^87^ Fitusiran is developed for the treatment of haemophilia A and rare bleeding disorders (RBD).^88^ Several more GalNAc–siRNA based therapeutics from Alnylam are in clinical trials.^89^

### Further development of multivalent GalNAc oligonucleotides

Alnylam Pharmaceuticals and Ionis (formerly Isis) Pharmaceuticals have had a strategic partnership in this area since 2004 and launched Regulus Therapeutics in 2007 as a joint venture to develop microRNA therapeutics – a variant of RNAi that uses or small hairpin loops called microRNA. Regulus Therapeutics developed an anti-microRNA conjugated to trivalent GalNAc ligand 13 for targeting miR-122 that is essential for Hepatitis C virus replication.^90–92^ Ionis Pharmaceuticals has taken the lead in the development of GalNAc-targeted antisense oligonucleotide (ASO) drug delivery to hepatocytes. Prakash *et al.* proved that ASO conjugates of GalNAc ligand 13 were 10-fold more potent than naked ASO in mouse experiments.^93^ The Alnylam linker with a prolinol group enables introduction of the trivalent GalNAc at the 3′-end of the nucleic acid.^81^ Østergaard *et al.* developed an efficient solution modification procedure to introduce trivalent ligand 13 at the 5′-end of an amine-modified oligonucleotide, and found GalNAc conjugated at 5′ end of the ASO was about 50% more potent than the 3′-end conjugate.^94^

**Fig. 7:**
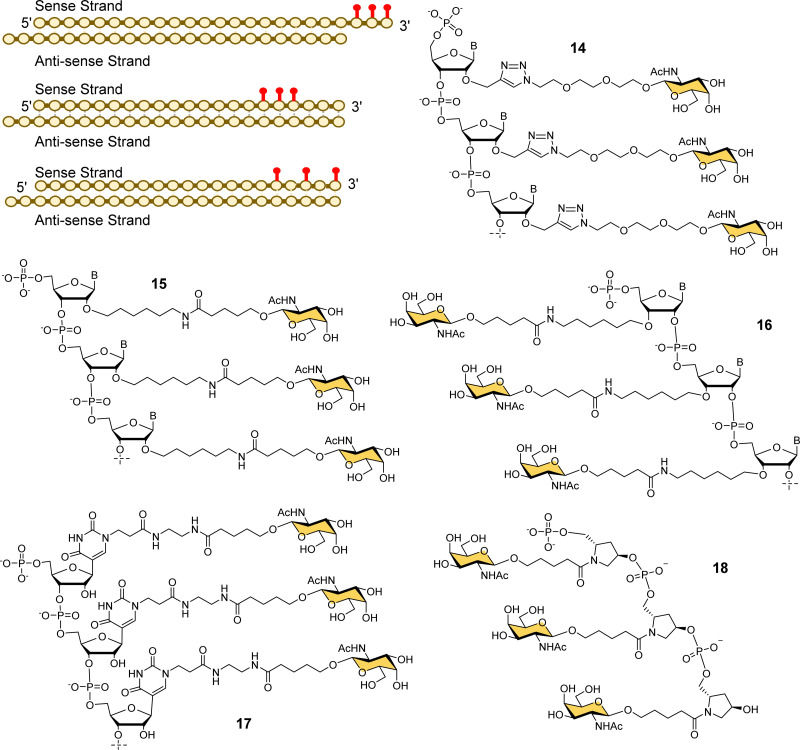
Various GalNAc–siRNA conjugates made by Alnylam Pharmaceuticals. The red dots represent some of the locations of GalNAc-derived nucleotides prepared.

Several companies have investigated strategies for constructing multivalent ligands from GalNAcylated phosphoramidite building blocks during solid phase oligonucleotide synthesis. This approach has the potential advantage of flexibly introducing the GalNAc residues at any position along the oligonucleotide chain, while maintaining the high coupling efficiencies that are typical of phosphoramidite-based oligonucleotide synthesis. For example, Matsuda *et al.*, at Alnylam Pharmaceuticals constructed a series of 3′-GalNAc–siRNA conjugates using oligonucleotide building blocks with GalNAc residues linked to either the 2′-position (Fig. 7, 14, 15) or 3′-position (16) on the ribose ring, or to N-1 of a pseudouridine base (17).^95^ Each type of trinucleotide was introduced at the 3′-end of the sense RNA strand, while the 2′-triazole-linked building blocks were also introduced systematically along the length of the sense RNA strand resulting in a trivalent clustered or dispersed display of GalNAc, as shown in Fig. 7. Most compounds showed similar *in vitro* and *in vivo* potencies to a siRNA targeted with the Alnylam ligand 13 at the 3′-end, however, potency was reduced for conjugates with the GalNAc residues towards the middle of the RNA sequence, and also when the GalNAc residues were spread out at the 5′-, 3′- and middle of the sequence. GalNAc-phosphoramidite building blocks derived from *trans*-4-hydroxyprolinol (18) have also been used to incorporate individual GalNAc residues to construct trimers at the 3′-end of the sense strand of siRNA oligonucleotides,^96^ and 5′-end of an ASO.^97^

**Fig. 8:**
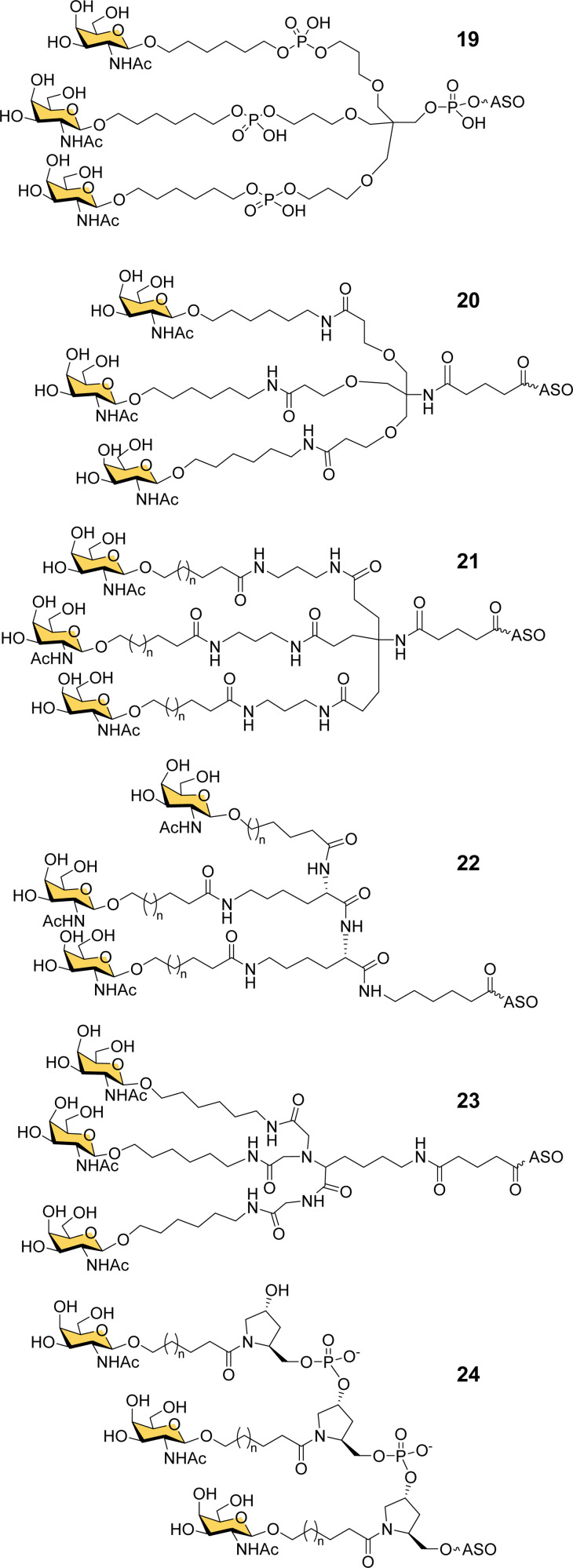
GalNAc clusters synthesized from six different classes of scaffolds.

Ionis scientists also investigated alternative strategies to introduce a trivalent GalNAc ligand at the 5′-end of oligonucleotides during solid phase synthesis. By using a tris-dimethoxytrityl-protected phosphoramidite building block derived from tetrahydroxymethylmethane, and a monovalent GalNAc phosphoramidite, it was possible to construct a phosphate-diester-linked GalNAc cluster (19, Fig. 8) on the solid support at the end of the oligonucleotide synthesis.^98^ An ASO designed to suppress SRB-1 expression had remarkably similar activity in mice whether conjugated to the new GalNAc cluster at its 5′-end or the Alnylam GalNAc ligand 13 at its 3′-end.^98^

Prakash *et al.* conducted a broad structure-activity study of triantennary GalNAc ligands coupled to ASOs that compared the Trebler and hydroxyprolinol phosphate diester ligands with a broad range of amide-linked clusters.^99^ They constructed 17 different branched or amino acid trivalent scaffolds, across five different classes of scaffold for synthesizing GalNAc clusters (Fig. 8, 19–24). Extensive *in vitro* and *in vivo* testing demonstrated that there were only small differences between the ability of different scaffolds to delivery ASOs effectively. Nevertheless, they noted 2- to 4-fold reductions in potency for longer hydrophobic linkers which was contrary to the design rules described earlier in this review. They identified a simplified tris-based ligand 20 (5′-THA-GN3) that could be synthesised more easily than the Alnylam compound 13, that they also noted shows potency in humans.^99^ Several Ionis GalNAc–ASO conjugates based on this ligand (Pelacarsen, Eplontersen, Donidalorsen and Olezarsen) are in phase three clinical trials.^100^ Many more GalNAc–ASO conjugates are in phase one and two clinical trials.^101^

**Fig. 9:**
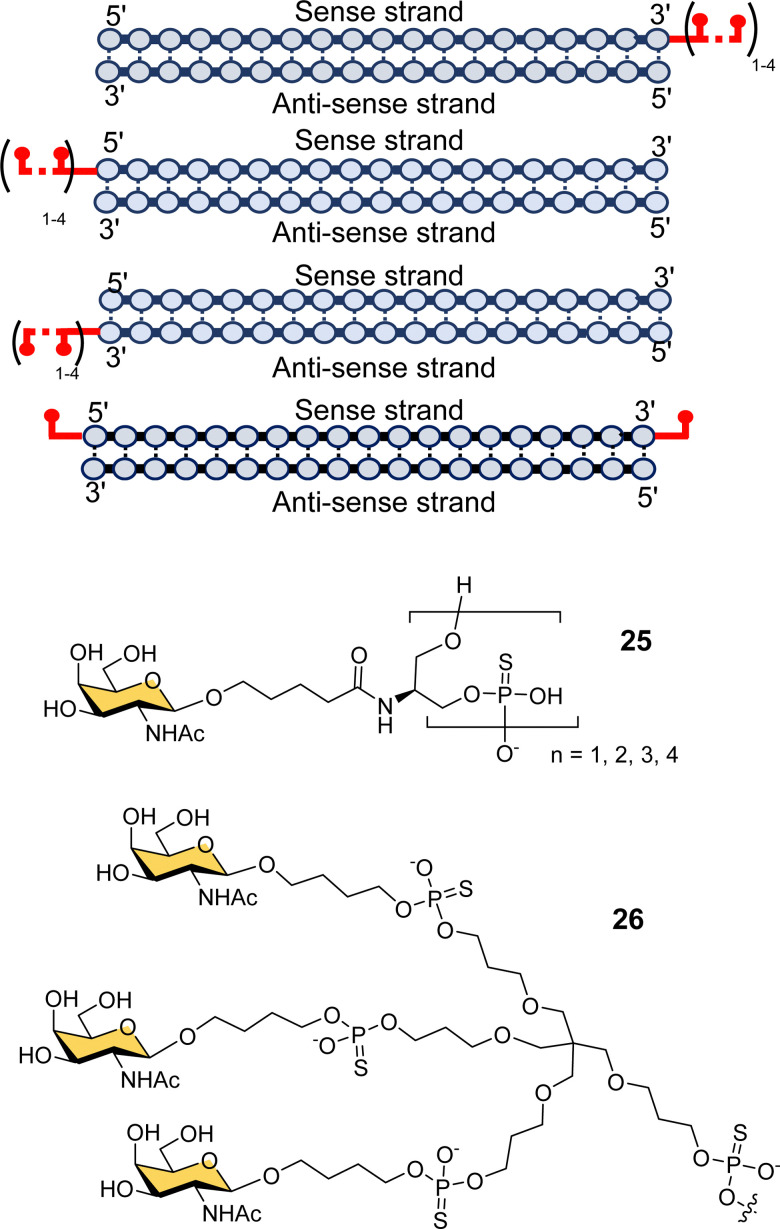
GalNAc–siRNA conjugates based on serinol-phosphorothioate repeat units introduced at 3′- and/or 5′-ends of siRNA.

Scientists at Silence Pharmaceuticals developed various serinol phosphorothioate-linked GalNAc siRNA conjugates.^102^ They had previously shown that capping 5′/3′ termini in 2′-OMe-RNA with serinol resulted in increased activity and serum stability over 24 h,^103,104^ and so investigated the use of GalNAcylated versions of this building block (25) for targeted delivery of siRNA (Fig. 9).^105^ Attaching between two and four GalNAc residues to either the 5′- or 3′-end of the sense strand, or to the 3′-end of the antisense strand gave similar activity to having triantennary glycan 26 at the 3′-end of the sense strand. Introducing any number of GalNAc residues at the 5′-end of the antisense strand led to the loss in function because of disruption in RNA-induced silence complex (RISC) formation that is central to the action of siRNA.^106^ Surprisingly, they found that introducing a single GalNAc on both the 3′- and 5′-ends of the sense strand for several different siRNAs exhibited better *in vivo* activity and longer duration of action in mice than siRNA with the triantennary GalNAc cluster.^105^ This is in contrast to the study by Matsuda *et al.* described above where they saw poor activity for siRNA with single GalNAc residues introduced at the 5′-end, 3′-end and middle of an siRNA sense strand.^95^ So, the Silence Therapeutics team hypothesised that using a serinol linker would influence siRNA stability after receptor-mediated uptake in hepatocytes, *i.e.*, along the endosomal–lysosomal pathway, and that this would result in more robust and more prolonged effects in *in vivo* experiments.^105^ Three siRNA–GalNAc conjugates from Silence Therapeutics, SLN-360 against lipoprotein A,^107,108^ SLN-124 against the hormone hepcidin,^109^ and SLN501 against complement C3 protein, are in phase 1 clinical trials.

**Fig. 10:**
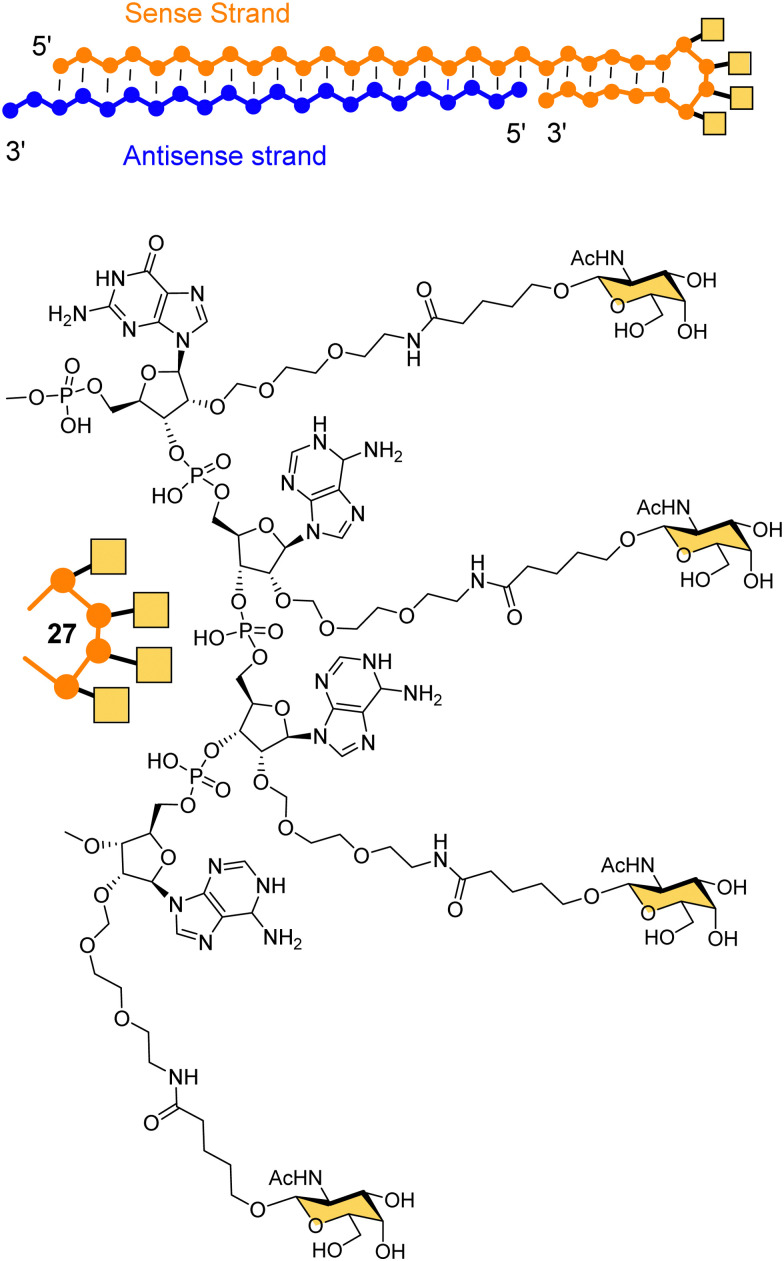
The chemical structure of GalXC with a tetravalent hairpin loop developed by Dicerna Pharmaceuticals.

Dicerna Pharmaceuticals developed a proprietary GalXc platform technology (Fig. 10).^110^ In this technology, the sense strand or Dicer-substrate RNA (DsiRNA) is a long strand having a ‘tetraloop’ hairpin region and a 21–23-mer antisense strand complementary to the target mRNA to be degraded.^111^ The self-complementary double stranded sequence forming the loop in the sense strand cannot easily be loaded by the RISC, which reduces off-target toxicity and only the antisense strand will be used for RNAi.^111^ GalNAc residues are attached on each of the four consecutive nucleotides of the tetraloop hairpin region (27) for recognition by the ASGP-R. Nedosiran,^112,113^ which is in phase three clinical trials, was developed using GalXC technology to treat primary hyperoxaluria by inhibiting expression of hepatic lactate dehydrogenase to prevent over production of oxalate. With the use of GalXC technology, five drug candidates are in different stages of clinical trials, and more than 20 are in the early stage of development or preclinical stage.^100^

**Fig. 11:**
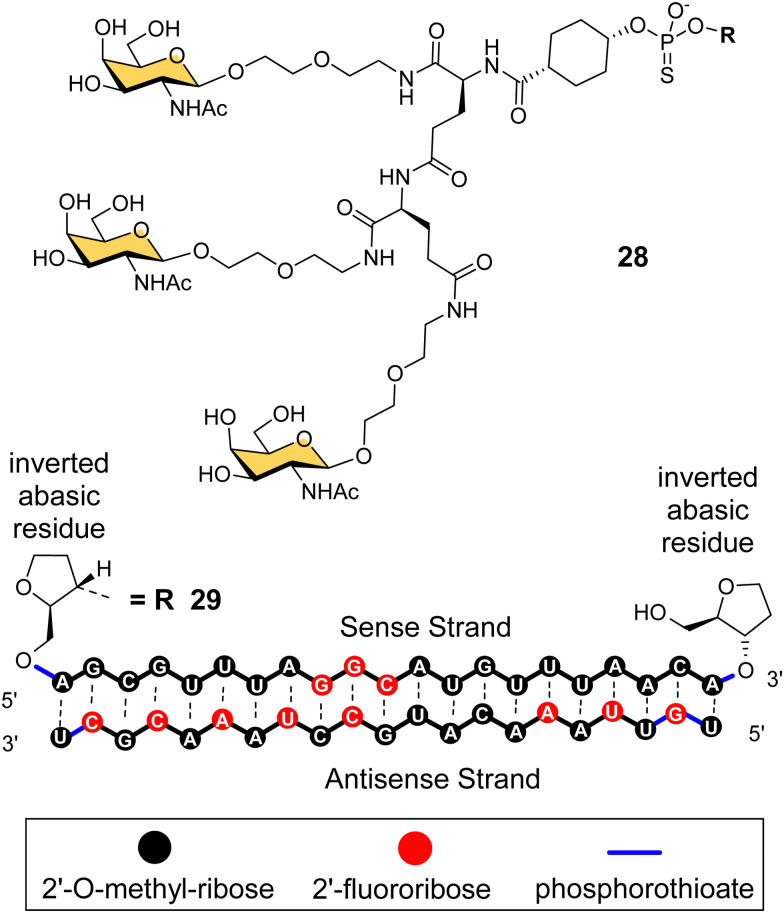
NAG37 GalNAc cluster 28 used by Arrowhead Pharmaceuticals in siRNA conjugates including ARO-AAT 29.

Arrowhead Pharmaceuticals have developed a GalNAc targeting agent for siRNA delivery as part of their TRiM™ (Targeted RNAi Molecule) delivery platform.^114^ Their trivalent GalNAc ligand (Fig. 11, ligand 28) is based on a γ-glutamyl dipeptide core that is reminiscent of the Lee YEE GalNAc cluster 4. This targeting group is being used in the development of a range of RNAi drugs including compound 29 for the treatment of alpha-1 antitrypsin (AAT) liver disease.^115^ The RNAi investigational therapeutic ARO-APOC3,^116^ targeting apolipoprotein C-III, is in stage three clinical trials. It is used to treat patients with hypertriglyceridemia, severe hypertriglyceridemia, and familial chylomicronemia syndrome.

### Other lectins for targeted delivery

Interest in using multivalent glycans for targeted delivery to the liver extends beyond delivery of oligonucleotides, to other cargoes including small molecule drugs^62,117^ and lysosome-targeting chimeras (LYTACs).^118^ It is beyond the scope of this review to acknowledge all those who have made contributions to this wider field. There are also many other human lectins that could be exploited in a similar way to ASGP-R,^119^ but so far only a relatively small number of them have been investigated for targeted delivery applications, and fewer for delivery of nucleic acids. Delivery to cells displaying human lectins that recognise mannose,^120^ mannose-6-phosphate,^121^ and sialic acid derivatives^122^ have been reviewed elsewhere, so here we will only highlight a few illustrative examples. There have been several reports of siRNA delivery to macrophage cells *via* the macrophage mannose receptor (CD206). These have included the use of mannosylated polymeric micelles,^123,124^ and polysaccharide nanoparticles.^125^ While there has not been much work so far in oligonucleotide delivery to other mannose-binding lectins such as DC-SIGN that is expressed on dendritic cells,^126^ DC-SIGN has been extensively investigated for delivery of antigens.^127^ As highly selective multivalent ligands for DC-SIGN have been developed,^128^ it is quite possible that targeted delivery of oligonucleotides using defined conjugates could be possible in the future. There is growing interest in the mannose-6-phosphate receptor for targeting to lysosomes.^121^ While the focus has been enzyme replacement therapies, LYTACS^129^ and delivery of Toll-like receptor ligands,^130^ delivery of antisense oligonucleotides into cells by this route has been reported.^131^ The development of ligands that can distinguish the members of the sialic acid-binding siglec family of receptors has also led to increased interest in their potential for targeted delivery. For example, liposomal nanoparticles have been targeted to macrophages,^132^ and eosinophils,^133^ using ligands for siglec-1 and siglec-8, respectively. There could be opportunities to apply similar systems for delivery of siRNA.

So far we have only considered targeting protein receptors with multivalent carbohydrate ligands, but as Fig. 1 shows, there are also important examples of targeted delivery of exogenous protein receptors that recognise cell-surface glycans. For example, bacterial toxins such as cholera toxin and shiga-like toxin have evolved to deliver toxic enzymes into cells by binding to specific glycolipids and instigating receptor-mediated endocytosis.^134^ The non-toxic B-subunit of cholera toxin (CTB) has long been used as a neuronal tracer;^135^ intra-muscular injection allows the protein to bind to its glycolipid ligand and enter motor neurones at the neuro-muscular junction, from where it undergoes retrograde trafficking to the cell body in the spinal cord or brainstem. Polylysine conjugates of CTB have previously been used for gene delivery applications,^136^ and the advent of well-defined bacterial toxin delivery vehicles,^137,138^ could also open possibilities for glycan-mediated delivery of nucleic acids beyond the blood–brain barrier.

## Conclusions

The use of various GalNAc-based technologies for targeting hepatocytes *via* the ASGP-R has been a remarkable success for the application of multivalent carbohydrates. Its origins can be traced back over 40 years to the early work of Y. C. Lee that laid the foundations for the field of multivalent glycoconjugates. High affinity trimeric ligands for ASGP-R and the preference for GalNAc over galactose dates back to the 1980s, as does knowledge of the distances between the ASGP-R binding sites that must be crosslinked for efficient affinity. The late 1980s and 1990s saw the first steps towards GalNAc-mediated delivery of nucleic acids, with another notable contribution from Lee and co-workers of the first defined nucleic acid conjugate in 1995.^59^ The key scaffold, that was to be later used in the Alnylam Pharmaceuticals drug Givosiran, was first described by Biessen and co-workers in 1999,^73^ and at its core, the 2-amino-2-(hydroxymethyl)-1,3-propanediol (tris buffer) unit, which has become a ubiquitous building block in multivalent glycoconjugates since Lee's first use of it in 1978.^20^ The importance of subsequent work of Alnylam Pharmaceuticals in developing GalNAc technology and taking it through clinical trials and into the clinic during the 2000s and 2010s cannot be overstated. GalNAc technology revived the promise of oligonucleotide-based drugs for clinical application and has become one of the most powerful approaches to delivering RNA-based cargos (siRNA and ASOs) to hepatocytes. The high efficacy, superior pharmacokinetics, low ectopic accumulation, and low RNAi-based toxicity improved RNAi-based drugs’ therapeutic window. Even the challenges of complex molecules such as these drugs *e.g.*, high cost and scale-up of production has not stood in the way of drugs reaching the clinic. Targeted delivery to hepatocytes is now a solved problem to such an extent that a 2021 UK Research and Innovation funding opportunity called “Early ideas to improve the delivery of nucleic acid therapeutics” explicitly stated that programmes focusing on delivery to hepatocytes were ineligible.^139^ It can be a long journey from fundamental research to impact in the clinic, and we are aware that, in trying to trace a concise history of GalNAc targeting, we have bypassed the work of many other glycoscientists who have exploited the ASGP-R for targeting hepatocytes. We hope that the success of GalNAc technology brings inspiration and confidence, especially for the pharmaceutical companies who can bring such technologies to the clinic, to invest further in the many other opportunities for using multivalent glycoconjugates or lectins for targeting different types of cells/tissues.

## Conflicts of interest

There are no conflicts to declare.

## Supplementary Material
